# Enhanced production of gamma‐aminobutyric acid in fermented carrot juice by utilizing pectin hydrolysate derived from pomegranate waste

**DOI:** 10.1002/fsn3.4282

**Published:** 2024-06-21

**Authors:** Dilara Devecioglu, Didem Kara, Rabia Tapan, Funda Karbancioglu‐Guler, Derya Kahveci

**Affiliations:** ^1^ Faculty of Chemical and Metallurgical Engineering, Department of Food Engineering Istanbul Technical University Maslak Turkey

**Keywords:** fermented carrot juice, gamma‐aminobutyric acid, *Levilactobacillus brevis*, pectin hydrolysate, prebiotic

## Abstract

In this study, a functional fermented beverage enriched with gamma‐aminobutyric acid (GABA) was produced. To achieve this, the prebiotic abilities of pectin obtained from pomegranate peel and its enzymatic hydrolysates were evaluated. Additionally, a functional fermented beverage enriched with GABA was produced by fermenting carrot juice with pectin hydrolysates. First, pectin was obtained at a yield of 8.91% from pomegranate peels. Pectinase‐catalyzed hydrolysis of the obtained pectin was applied using different enzyme concentrations and hydrolysis times, and the effect of these hydrolysates on the growth of *Levilactobacillus brevis* was determined. Although the Fourier transform infrared (FT‐IR) spectra of the resulting hydrolysates were similar, their degree of esterification compared to that of pectin was statistically different (*p* < .05). Considering the viability analysis and GABA production of *L. brevis* in the liquid medium supplemented with pectin or its hydrolysate, the hydrolysate obtained by treatment with 400 μL enzyme for 2 h and having a high glucose content (216.80 mg/100 g) was selected for application in fermented carrot juice. During fermentation (24, 48, and 72 h), a remarkable change was observed, especially in the amounts of lactic acid and malic acid, while the amount of GABA in carrot juice varied between 25 and 46 mg/mL and increased with the increase in hydrolysate concentration. It was observed that the total phenolic content and antioxidant activity of carrot juice were highly affected by the hydrolysate concentration. This study demonstrated that pectin hydrolysate obtained from food waste could be a potential prebiotic and could be used in the production of a functional beverage with improved GABA content.

## Graphical abstract

Pectin extracted from industrial pomegranate waste was enzymatically hydrolyzed and applied as a prebiotic in carrot juice fermentation. Added pectin hydrolysate improved bioactive components’ concentrations, i.e., phenolic compounds, organic acids, and gamma‐aminobutyric acid.

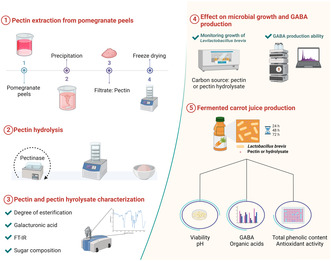

## 1 INTRODUCTION

Consumer interest in functional foods has been on the rise for the past few decades, experiencing an increase in such foods in the post‐pandemic era due to the positive effects of bioactive components used in functional food formulations on immune health (Singh, Tripathi et al., 2020; Wan‐Mohtar et al., 2022). Functional foods exist in various forms, such as cereal‐based, dairy, meat, bakery products, confectionery, ready‐to‐eat meals, etc. (Aryee & Boye, 2014). Functional beverages (FBs), among others, are the leading category with a fast growth rate due to the ease of formulation and development of new products with a variety of bioactive components, easier storage and delivery of refrigerated products, and consumer satisfaction in terms of container convenience, size, and shape (Corbo et al., 2014). Fermented functional beverages (FFBs), in particular, which have been produced and consumed since ancient times, have gained interest due to consumer demand (Tang et al., 2023). Such beverages provide reduced sodium and salt, as well as improved gut health, both of which have been the major reasons for consumers’ interest in FFBs (Ruiz Rodríguez et al., 2021). According to FAOSTAT (2023), the global production of FFBs has increased from 26.7 million tons in 2010 to 280.3 million tons in 2020. Fermentation by lactic acid bacteria (LAB) is a sustainable and feasible approach to improve the nutritional and sensory properties of FB (Marsh et al., 2014). In fermented foods, LAB primarily convert carbohydrates into lactic acid to increase shelf life and microbial safety (Hu et al., 2022). Furthermore, the evaluation of LAB as a Generally Recognized as Safe (GRAS) organism raises their importance from the perspective of both consumers and producers (Cai et al., 2019; Cataldo, Villegas, et al., 2020).

The leading category in FFB involves dairy‐based products; however, the need for new formulations for lactose‐intolerant consumers, as well as the developing vegan products category with increased demand, has led the market to focus on fruit‐based FFB (FFFB) production (Keșa et al., 2021; Ruiz Rodríguez et al., 2021; Vodnar et al., 2019). Fruits present an ideal medium for fermentation since they already contain valuable ingredients, such as carbohydrates, vitamins, and minerals. Since around half the weight of fruits produced ends up in waste during transportation and processing, recent interest in FFFB production involves the use of such waste together with/instead of the fruit itself (Alexandre et al., 2023; Ruiz Rodríguez et al., 2021; Vodnar et al., 2019). For example, Chinese bayberry pomace was fermented using a mixed culture of yeast, LAB, and acetic acid bacteria (Zhu et al., 2022); pineapple by‐products were fermented by *Saccharomyces cerevisiae* (Aguilar, 2022; Hien et al., 2022). As for the fruit waste or its extract added to the fermentation medium, examples include a kombucha‐like (Leonarski et al., 2021) or a soy‐based (Vieira, Battistini, et al., 2021; Vieira, de Souza, et al., 2021) FFB with acerola by‐products' extract, and an apple‐based FFFB with polysaccharides extracted from prickly pear (Álvarez et al., 2021).

Probiotics are live microorganisms that exert potential health benefits, especially improved gut health, immunomodulatory activity, cholesterol‐lowering effect, and prevention of liver diseases and certain types of cancer, to the consumer when administered in adequate amounts (Das et al., 2022). While probiotic microorganisms must be evaluated according to many criteria, the probiotic characteristics of LAB have been studied for decades (Abushelaibi et al., 2017). Most commonly used probiotic LAB are from *Lactobacillus* genera (Diez‐Gutiérrez et al., 2020). These positive and remarkable effects of probiotic microorganisms on health, as well as the increasing tendency of consumers toward healthier foods, are presented as a reason for the emergence of more sustainable and natural ingredient‐containing foods (Ayivi et al., 2020). Therefore, probiotic FFBs have been recently emphasized as an upcoming trend in FFB research, with several examples on the global market already widespread (Ruiz Rodríguez et al., 2021).

Gamma‐aminobutyric acid (GABA) is a non‐protein amino acid that is widely found in plants, animals, and microorganisms (Ramos‐Ruiz et al., 2018). Its function depends on the host: It acts as the primary inhibitory neurotransmitter in the central nervous system (CNS) of animals (Ngo & Vo, 2019), whereas it is involved in protection against plant stress (Kinnersley & Turano, 2000). Instead of extraction from natural sources, enzymatic or chemical synthesis, production of GABA through fermentation has been preferred due to several advantages, including the use of a sustainable fermentation medium as the starting material and green production at mild conditions with reduced environmental effects (Luo et al., 2021). Therefore, GABA is now considered as an important postbiotic with increasing research accumulating in the field, emphasizing GABA's positive effects on mood modulation, sleep improvement (Hepsomali et al., 2020), epilepsy (Feng et al., 2022), depression (Luscher et al., 2011), and several neural diseases (Xu et al., 2020; Xu & Wong, 2018). Functional food research has joined the trend and several probiotic, GABA‐enriched FFBs have been reported, including FFFBs (Cataldo, Villena, et al., 2020; Kantachote et al., 2017; Kim et al., 2009; Liu et al., 2022; Nakatani et al., 2022; Sun et al., 2022; Zhou et al., 2019) as well as dairy‐based (Zarei, 2020) and legume‐based (Fan et al., 2023; Li et al., 2016; Lorusso et al., 2018; Pontonio et al., 2020; Song & Yu, 2018).

Plant cell walls include a structural polysaccharide called pectin, which is a high‐molecular‐weight macromolecule (Freitas et al., 2021). Pectin has been shown to provide health and technical advantages in several fields (Moslemi, 2021). For the species composition of gut microbial communities and their metabolic outputs to be positively impacted, it is crucial to include non‐digestible polysaccharides like pectin in the diet (Chung et al., 2017). While plant food products are sources of pectin, an increasing number of studies on the evaluation of food waste have shown that these sources can also be used to obtain pectin (Dranca & Oroian, 2018).

Pectin has traditionally been extracted from industrial fruit waste, such as citrus and potato peel, apple pomace, and sugar beet pulp (Singh & Tingirikari, 2021). Food industry uses pectin in several products due to its thickening and gelling properties. Additionally, pectin has been shown to have prebiotic effects, which made it an ingredient for functional food formulations as well (Ferreira‐Lazarte et al., 2018; Foti et al., 2022; Ho et al., 2017; Kuo et al., 2021; Larsen et al., 2018; Liu et al., 2021; Singh, Prakash et al., 2020; Wilkowska et al., 2021; Yeung et al., 2021; Yu et al., 2022; Zhu et al., 2019). Both the degree of esterification (Foti et al., 2022; Larsen et al., 2018; Yeung et al., 2021) and the individual concentrations of monosaccharides (Ai et al., 2022; Ferreira‐Lazarte et al., 2018; Sabater et al., 2021; Yeung et al., 2021; Yu et al., 2022; Zhu et al., 2019) were reported to have an effect on the prebiotic potential of pectin. Furthermore, modifications to the structure of the pectin macromolecule may confer additional health benefits to pectin, as well as being prebiotic (Lu et al., 2024).

The objective of the present study was to produce a probiotic FFFB via *Levilactobacillus brevis* fermentation of carrot juice. Pectin extracted from pomegranate waste, as well as its hydrolysates produced by enzymatic hydrolysis, were added to the medium as prebiotics. The viable LAB count and the bioactivity of the final product were investigated in terms of the GABA content, phenolic compounds’ concentration, and antioxidant capacity.

## 2 MATERIALS AND METHODS

### 2.1 Materials

Pomegranate waste was supplied from industrial fruit juice producer Dimes Gıda Sanayi ve Ticaret A. Ş. (Türkiye). *L. brevis*, previously isolated from sourdough (Demirbaş et al., 2017), was used in this study. The stock culture was preserved in 50% glycerol solution at −80°C. Pectinase for enzymatic hydrolysis of pectin was obtained from Novozymes (Denmark). All growth media (1.10660 and 1.10661) were supplied from Merck (Darmstadt, Germany). Solutions and growth media were prepared using the double‐distilled water. The organic solvents and other chemicals used were of HPLC or analytical grade supplied from Sigma‐Aldrich (St Louis, MO, USA) and Merck (Darmstadt, Germany). Monosodium glutamate (MSG) (030921‐ALF‐885) was obtained from Alfasol (Türkiye).

### 2.2 Methods

#### 2.2.1 Chemical composition of pomegranate waste and pectin extraction

The approximate composition of the pomegranate waste (moisture content, lipid, ash, and protein content) was determined according to the method described by the Association of Official Analytical Chemists (AOAC, 1997). Dry matter was determined by drying samples to constant weight at 102 ± 3°C. Total ash was determined by calcination in the muffle furnace at 500°C, until a constant weight was achieved. The total nitrogen concentration was obtained using the Kjeldahl method, and the protein concentration was estimated using a nitrogen conversion factor of 6.38. The oil content was determined by Soxhlet extraction with hexane at the solvent's boiling point.

Pomegranate waste was stored at −20°C, lyophilized (Christ, Harz, Germany), and ground (Fakir, Germany). The extraction of pomegranate peel pectin was carried out with citric acid solution (1:100 w/v) in a water bath (Memmert, Germany) using extraction conditions of 60 min, 80°C, and a solid–liquid ratio of 3:100 (g/mL) under stirring by the modified method of Pereira et al. (2016). After the mixture was cooled to room temperature, it was centrifuged (4000 rpm, 25 min, 4°C) and the supernatant was filtered. The same volume of ethanol (96% w/w) was added to the filtrate for pectin precipitation, and the obtained mixture was kept at 4°C for 1 h and centrifuged (7 min, 4°C, 4000 rpm). The collected pellet was washed with ethanol, separated using a sieve, and ground after lyophilization. The gravimetric yield (%) was estimated as the ratio between the weight of the powdered pectin and the weight of the powdered pomegranate waste (g/g, x100), both on a dry basis.

#### 2.2.2 Pomegranate waste pectin hydrolysis

The extracted pectin was mixed with distilled water (20 mg pectin/mL distilled water) and dissolved using an Ultra‐Turrax (IKA T25 digital, Germany). The pH was adjusted to 4.0 with 1 M sodium chloride (NaOH). Pectinase‐catalyzed hydrolysis was conducted at 50°C for different reaction times by varying enzyme concentrations (Table 1) in a shaking water bath. At the end of the hydrolysis process, the enzyme was inactivated by boiling at 100°C for 10 min. After cooling to room temperature, the mixture was centrifuged (4000 rpm, 7 min, 4°C), and ethanol (96% w/w) was added to the supernatant (1:1 v/v). The final mixture was stored for 1 h at 4°C, after which the supernatant was separated, concentrated, and lyophilized (Zhai et al., 2018).

**TABLE 1:** Conditions of pectin hydrolysis.

	P2H2	P2H4	P4H2	P4H4
Enzyme (μL/g pectin)	200	200	400	400
Reaction time (h)	2	4	2	4

#### 2.2.3 Characterization of pectin and pectin hydrolysates

Fourier Transform Infrared Spectroscopy (FTIR; Bruker Tensor II, Massachusetts, USA) spectra were collected from the extracted pectin, its hydrolysates, and commercial pectin in the frequency range of 400–4000 cm^−1^.

Monosaccharide composition analysis was performed using the instructions given for Waters amide column with slight modifications. Briefly, 1 mL of 4 M trifluoroacetic acid (TFA) was added to a 4 mg sample and hydrolyzed for 4 h in an oven at 110°C. After the hydrolysis, the samples were cooled to room temperature, neutralized with a strong base (4 M NaOH, pH 7.0), and filtered (0.45 μm) before analysis. The HPLC (high‐performance liquid‐chromatography) analysis was carried out with a Waters 2690 HPLC system (Waters Corporation, Milford, MA, USA) equipped with a differential refractive index detector (Waters 2140). The isocratic separation was achieved on a carbohydrate amino (NH_2_) column (Waters, 300 mm × 3.9 mm, 10‐μm particle size) with 1 mL/min flow rate of acetonitrile–water–acetic acid (99:99:2, v/v/v) as a mobile phase.

The galacturonic acid (GalA) content of pectin and its hydrolysates was determined using the method of Blumenkrantz and Asboe‐Hansen (1973) with slight modifications. The pectin and hydrolysates (5 mg), 2 mL of 72% sulfuric acid (H_2_SO_4_), and 15 mL of distilled water were mixed for 1 h at room temperature. Then, 0.5 mL of the sample was taken and mixed with 3 mL of concentrated H_2_SO_4_ including 12.5 mM sodium tetraborate. The mixture was kept in boiling water for 5 min and then cooled immediately in an ice‐water bath. Next, 50 μL of 0.15% 3‐phenylphenol reagent was added and kept at room temperature for 10 min. The absorbance was read at 520 nm (BioTek Technologies, Winooski, VT, USA). The GalA content was determined using the standard curve of 100–600 nmol/mL GalA solutions.

The titrimetric method of Hosseini et al. (2016) was modified to determine the degree of esterification (DE) of pectin and hydrolysates. Briefly, the sample (75 mg) was dissolved in 100 mL of distilled water. After complete dissolution, 5 drops of phenolphthalein reagent were added, and the sample was titrated with 0.1 M NaOH (*V*_1_, mL). After 20 mL of 0.5 M HCl was added, the sample was kept at room temperature for 15 min and mixed until the pink color disappeared. After adding 5 drops of phenolphthalein reagent, the mixture was titrated with 0.1 M NaOH until a slight pink color was observed (*V*_2_, mL). DE (%) was calculated according to Equation (1).

(1) $$\mathrm{DE}\;\left(\%\right)=V_{2}/\left(V_{1}+V_{2}\right)\times 100$$

#### 2.2.4 Effect of pectin on the growth of *L. brevis* and GABA production

The stock culture of *L. brevis* at −80°C in glycerol was grown in de Man, Rogosa and Sharpe liquid medium (MRS broth; Merck, Darmstadt, Germany) and inoculated to MRS agar (Merck, Darmstadt, Germany) for further analysis. *L. brevis* on MRS agar were inoculated into MRS broth and incubated at 37°C for 24–48 h (N‐Biotek, Korea).

A modified MRS medium supplemented with 4% w/v pectin or its hydrolysates instead of glucose, and 2% monosodium glutamate was used to investigate the effect of pectin and its hydrolysates on the viability and GABA production potential of *L. brevis*. The modified MRS medium (g/L) was prepared by adding peptone from casein, 10; meat extract, 8; yeast extract, 4; dipotassium phosphate (K_2_HPO_4_), 2; Tween 80, 1; di‐ammonium hydrogen citrate, 2; sodium acetate, 5; magnesium sulfate (MgSO_4_), 0,2; manganese sulfate (MnSO_4_), 0,04; and monosodium glutamate, 20. The pH of the medium was adjusted to 4.5 with 0.5 M citric acid. Pectin and its hydrolysates were dissolved in 25 mL of the prepared medium with the help of Ultra‐Turrax and sterilized by filtration before addition to the autoclaved medium through 0.45‐μm filters (Sartorius, Germany). The stock culture of *L. brevis* was grown in de Man, Rogosa and Sharpe liquid medium (MRS broth; Merck, Darmstadt, Germany). After incubation, bacterial cell concentration was adjusted to approximately 10^8^ CFU (colony‐forming units)/mL by measuring turbidity (Den‐1B McFarland BioSan Densitometer, Latvia). Then, the bacterial culture (1%) was inoculated into 100 mL of the prepared medium, and all media were incubated at 37°C for 72 h at 100 rpm.

After fermentation, LAB counts were determined by using the pour plate technique on MRS agar under anaerobic incubation at 37°C for 24 h. The counts were expressed as CFU/mL. For GABA analysis, the remaining medium was centrifuged (10,000 rpm, 4°C, 10 min), and the supernatant was filtered through a 0.45‐μm filter. Then, 2 mL of the supernatant was mixed with 8 mL of 75% ethanol, shaken (Janke & Kunkel, Germany) for 15 min at room temperature, and centrifuged at 4000 rpm, at 4°C, for 15 min (Eppendorf 5804 R, Germany). The supernatant was filtered through a 0.45‐μm filter and stored at −18°C until HPLC analysis.

The GABA production potential of the microorganism was determined by the method of Gao et al. (2022) with the Agilent Technologies 1100 HPLC system consisting of a G1379A degasser, a G1311A quaternary pump, a G1313A ALS autosampler, and a G1316A TCC column heater using a C18 column (Supelco, USA, 250 mm × 4.6 mm, 5 μm) at 40°C. HPLC‐grade water containing 0.1% phosphoric acid (A) and 100% methanol (B) was used in isocratic flow (97:3, v/v). The flow rate was set at 0.70 mL/min. The detection was done with a diode array detector (DAD) (Agilent G1315D) at 210 nm. The standard curve was prepared with GABA standard in the concentration of 0.5–10 mg/mL.

#### 2.2.5 FFB production by fermentation of carrot juice

Carrot juice containing P4H2, the pectin hydrolysate selected according to the results of viability and GABA analysis, was fermented with *L. brevis*. Briefly, carrots purchased from the market were crushed using a juicer. MSG (2%, w/v) was added to the carrot juice to induce GABA production, and the pH was adjusted to 4.5 using 0.5 M citric acid or 0.1 M sodium bicarbonate. The prepared carrot juice (50 mL) was pasteurized in a water bath at 65°C for 30 min (Andrade et al., 2019; Kim et al., 2009). Then, P4H2 (1, 2, 4%) was added and mixed at 7000 rpm with Ultra‐Turrax. The carrot juice was inoculated with *L. brevis* to a final concentration of 10^6^ CFU/mL and incubated at 37°C, 100 rpm, 72 h. Samples were taken at 24‐h intervals during incubation for further analysis.

The pH of the collected samples and the viability of *L. brevis* during incubation were determined. The GABA content of samples (without extraction) was determined as mentioned above. The conducted GABA method was modified to determine other organic acid contents in the samples. The flow rate and column temperature were set at 0.5 mL/min and 30°C, respectively, and an isocratic flow (0.1% phosphoric acid:100% methanol, 97.5:2.5, v/v) was used.

#### 2.2.6 Total phenolic content and antioxidant activity of FFBs

The phenolic compounds in the carrot juice samples during fermentation were extracted according to the method of Capanoglu et al. (2008) with slight modifications. First, samples were weighed (0.75 g) and mixed with 3.75 mL of 75% methanol. After sonication (Bandelin Sonorex, Germany) for 15 min, the samples were centrifuged (4000 rpm, 4°C, 10 min). The collected supernatant was stored at −18°C until analysis.

The extracts’ total phenolic contents (TPCs) were determined by the Folin–Ciocalteu method modified from Velioglu et al. (1998) using gallic acid (GA) as a standard. The extract (100 μL) was mixed with 0.75 mL Folin–Ciocalteu reagent and 0.60 mL of 7.5% sodium bicarbonate. After keeping the mixture for 90 min at room temperature, the absorbance was read at 765 nm. Results were expressed in milligrams (mg) of gallic acid equivalent per 100 grams of dry weight ((GAE)/100 g dw) of sample. The total antioxidant capacities (TACs) of the sample extracts were evaluated by the copper‐reducing antioxidant capacity (CUPRAC) and the 1,1‐diphenyl‐2‐picrylhydrazyl (DPPH) methods (Apak et al., 2004; Kumaran & Karunakaran, 2006) and the results were expressed in milligrams (mg) of Trolox equivalents per 100 g of dry weight ((TE)/100 g dw) of sample.

#### 2.2.7 Statistical analysis

All results were reported as mean ± standard deviation of three independent replicates with three parallel measurements. The statistical significance of differences among groups was evaluated by factorial analysis of variance (ANOVA) followed by Tukey's test using Minitab (Version 18.0, USA), and significance was identified with a value of *p* < .05.

## 3 RESULTS AND DISCUSSION

### 3.1 Characterization of pectin and its hydrolysates

In the pomegranate waste samples, crude protein, ash, moisture, fat, and total sugar contents were 0.92 ± 0.01%, 1.13 ± 0.03%, 67 ± 0.01%, 0.63 ± 0.01%, and 30.13 ± 0.02%, respectively. The extraction yield of pectin (%) was 8.91 ± 1.67, which is in the range of previous studies (Pereira et al., 2016; Yang et al., 2018). Then, pectin was enzymatically hydrolyzed in four different conditions, as shown in Table 1. All hydrolysates and pectin were evaluated in terms of galacturonic acid (GalA), degree of esterification (DE), and monosaccharide composition (Table 2). The DE of pectin and its hydrolysates varied between 34.36% and 49.90%, indicating that the produced fractions were in the low methoxyl form (DE lower than 50%) (Santos et al., 2017). The polymer has to include at least 65% GalA to be classified as commercial pectin (Guandalini et al., 2019), and even if the results were lower, it is known that extraction conditions might have an impact on GalA content (Oliveira et al., 2016). After hydrolysis, a statistically significant difference was determined between the DE values; however, no noticeable effect of hydrolysis on GalA content was detected.

**TABLE 2:** Characterization of pomegranate pectin and its hydrolysates.

Sample	DE (%)	GalA (%)	Monosaccharide composition (mg/100 g)	
			Glucose	Rhamnose
Pectin	49.90 ± 2.69^a^	30.25 ± 3.76	279.80 ± 86.10^a^	1599.00 ± 184.00
P2H2	36.34 ± 7.73^b^	32.94 ± 2.38	59.60 ± 68.80^b^	1838.30 ± 108.30
P2H4	34.36 ± 2.40^b^	29.50 ± 1.53	–^1^	2002.00 ± 377.00
P4H2	37.17 ± 2.05^b^	26.82 ± 2.90	216.80 ± 66.50^a^	1659.76 ± 17.95
P4H4	34.49 ± 2.47^b^	30.92 ± 3.23	50.50 ± 58.30^b^	1794.00 ± 25.70

*Note*: ^a,b^Different superscripts within the same column indicate statistically significant differences (*p* < .05).

^1^ Not detected.

The main monosaccharides in the hydrolysates were rhamnose and glucose, similar to pectin, except for P2H4. Glucose concentrations in three hydrolysates were significantly lower than in intact pectin, whereas P4H2 was enriched in glucose. Some monosaccharides might undergo a degradation during the extraction as mentioned by Garna et al. (2007) who observed that the variation in monosaccharide content may be due to the alcohol precipitation.

**FIGURE 1:**
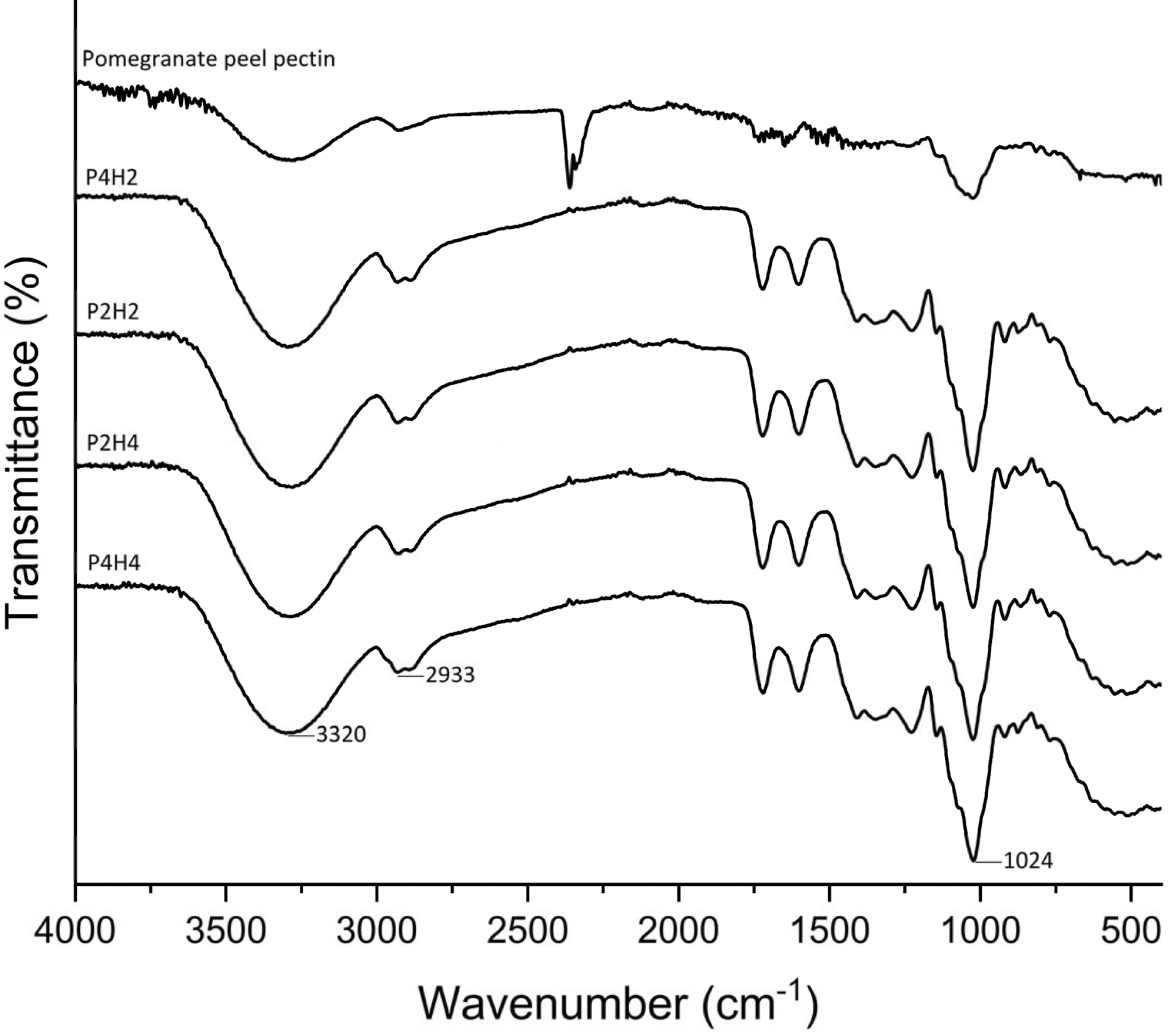
Fourier transform infrared spectroscopy (FT‐IR) spectrum of pectin and its different hydrolysates.

In Figure 1, the FT‐IR spectrum of pectin and its four different hydrolysates was visualized in the range of 400–4000 cm^−1^. As seen, there are two main bands between 3600 cm^−1^ and 2500 cm^−2^. The first band at around 3320 cm^−1^ corresponds to the absorption caused by hydroxyl (OH) stretching due to intermolecular and intramolecular hydrogen bonds (Yang et al., 2018), and the absorption at the OH site for pectin is due to the intermolecular and intramolecular hydrogen bonds of the single‐bond galacturonic acid backbone. The second peak, centered at approximately 2933 cm^−1^, represents CH single‐bond absorption, which includes CH, CH_2_, and CH_3_ stretching and bending vibrations. The region between 1800 and 1300 cm^−1^ is critical because of providing information about the degree of esterification due to the presence of the ester functional group and carboxylic acid peaks in this region.

The ester carbonyl's carbon monoxide (CO) stretching vibration may be responsible for the strong absorption band at 1731 cm^−1^, whereas the unmethylated carboxyl group's CO stretching vibration was responsible for the absorption peak at 1603 cm^−1^. The “fingerprint” area for pectin is thought to be fairly strong absorption patterns below 1500 cm^−1^, and they cannot be definitively attributed to any specific vibration (Abid et al., 2017). Therefore, Florinela et al. (2010) reported that glucose, fructose, and sucrose showed intense and characteristic bands in the region between 1200 and 900 cm^−1^, and the peaks were recorded at 1033, 1063, and 995 cm^−1^, respectively. There are peaks around 1031 and 1024 cm^−1^, which might indicate the presence of glucose in the samples.

### 3.2 Effect of pectin on the growth of *L. brevis* and GABA production

To stimulate the probiotic microorganism growth, the functional effect of prebiotics including pectin is crucial (Hurtado‐Romero et al., 2021). It has been shown that pectin hydrolysates obtained by different methods have a prebiotic effect. The prebiotic effect of citrus pectin obtained by the enzymatic method has been demonstrated (Ho et al., 2017; Lu et al., 2024). Therefore, the prebiotic abilities of pectin and its hydrolysates for *L. brevis* were tested in a medium free from glucose for 72 h incubation. In comparison to the control group, which includes glucose as a carbon source, hydrolysates other than P2H4 were shown to result in the greatest viability, whereas there was no significant difference in GABA production between potential prebiotics (Table 3). Under conditions containing 14.96% whey powder and a higher amount of MSG (4.95%) than the current study, the amount of GABA produced by *L. brevis* A3 was much lower (Alizadeh Behbahani et al., 2020). Even if the increase in viability may vary based on the microorganism, it has been noted that the pectin obtained from citrus peel affected the viability of *Lactobacillus paracasei* LPC‐37 depending on the pectin obtaining method. At the end of the 48 h incubation, the increase in cell density was between 1.17 and 2.85 log CFU/mL for pectin obtained with different methods (Zhang et al., 2018).

**TABLE 3:** The prebiotic and GABA production effect of pomegranate pectin and its hydrolysates.

Sample	Viability log increase (CFU/mL)	GABA concentration (mg/mL)
Pectin	2.51 ± 0.51^ab^	20.86 ± 7.15
P2H2	2.73 ± 0.75^ab^	24.23 ± 14.55
P2H4	1.75 ± 0.59^b^	22.45 ± 6.35
P4H2	2.42 ± 0.65^ab^	23.80 ± 4.48
P4H4	2.67 ± 1.04^ab^	23.69 ± 6.90
Control (glucose)	3.89 ± 0.38^a^	0.00 ± 0.00

*Note*: ^a,b^Different superscripts within the same column indicate statistically significant differences (*p* < .05).

Among all samples, the ability of P2H4 to preserve *L. brevis* viability during incubation was the lowest. It was shown that *L. brevis* utilize glucose (Zhang & Vadlani, 2015) and cannot mostly use rhamnose as a carbon source (Neveling et al., 2012), therefore the deficiency of P2H4 on viability may be because it does not contain glucose. On the other hand, P4H2 had the highest glucose concentration among the hydrolysates (Table 2) and performed similarly with the others in terms of viability and GABA production (Table 3). Considering the shorter reaction time applied to produce this sample together with the aforementioned findings, P4H2 was selected for future tests.

### 3.3 Production of FFB enriched in GABA

Carrot juice containing pectin hydrolysate (P4H2) and MSG at different concentrations was fermented with *L. brevis* to obtain FFB samples, which were evaluated in terms of viability, GABA production, total phenolic content, and antioxidant activity during fermentation (at 0, 24, 48, and 72 h). The effect of MSG, which is the substrate for GABA production by microorganisms (Park et al., 2021), was found to be statistically significant for GABA concentration (*p* < .05), which was drastically low in FFB samples where MSG was not added (data not shown); therefore, all analyses were completed for samples including MSG at 2% w/v concentration.

**FIGURE 2:**
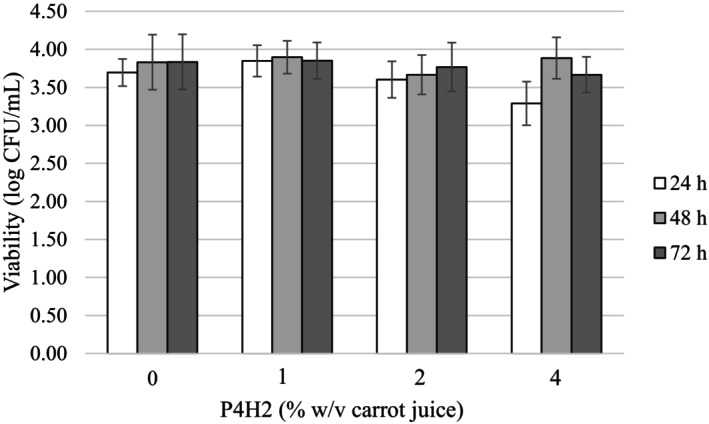
*Levilactobacillus brevis* viability throughout fermentation.

As expected, it is possible to observe a decline in the pH of the medium due to microbial growth during fermentation (Sharma & Mishra, 2013). Since the pectin hydrolysate in FFB had no adverse effect on viability (Figure 2), the pH decrease was observable for 24, 48, and 72 h fermentation as shown in Table 4. Fermentation was initiated by inoculating carrot juice with pH value adjusted to 4.5, and a decrease to 3.80–3.92 values was observed within 72 h.

**TABLE 4:** pH change throughout fermentation.

P4H2 (w/v carrot juice)	Time (h)		
	24	48	72
0	4.28 ± 0.10^a,x^	4.04 ± 0.10^b,x^	3.92 ± 0.08^c,x^
1	4.30 ± 0.04^a,x^	4.08 ± 0.03^b,x^	3.96 ± 0.08^c,x^
0.02	4.20 ± 0.04^a,x^	4.05 ± 0.06^b,x^	3.93 ± 0.05^c,x^
0.04	4.05 ± 0.05^a,y^	3.89 ± 0.05^b,y^	3.80 ± 0.08^c,y^

*Note*: ^a–c^Different superscripts within the same row indicate statistically significant differences (*p* < .05). ^x,y^Different superscripts within the same column indicate statistically significant differences (*p* < .05).

The primary catabolic end product produced by LAB from carbon sources is lactic acid (König & Fröhlich, 2017), and it is possible to detect lactic acid in the fermented product. Herewith, six different organic acids (tartaric acid, ascorbic acid, lactic acid, acetic acid, citric acid, and malic acid) were monitored in FFB samples (Table 5). During fermentation, lactic acid was mostly produced. Lactic acid plays an important role in the nutritional value of fermented products (Kun et al., 2008), and its concentration reached to 2.29 mg/mL after 72 h. The concentration of lactic acid in carrot juice fermented by two different strains of *Bifidobacterium* for 20 h was around 15 mg/mL (Kun et al., 2008), which is higher than the present study. However, this might be due to the type of microorganism that may use different compounds during fermentation.

**TABLE 5:** Organic acid content of FFBs.

P4H2 (%, w/v carrot juice)	Lactic acid (mg/mL)			Malic acid (mg/mL)			Acetic acid (mg/mL)			Citric acid (mg/mL)		
	Time (h)			Time (h)			Time (h)			Time (h)		
	24	48	72	24	48	72	24	48	72	24	48	72
0	1.10 ± 0.38^y^	1.05 ± 0.11^y^	1.09 ± 0.38^y^	n.d.	0.04 ± 0.01^β^	0.08 ± 0.06^β^	0.71 ± 0.20^a^	0.20 ± 0.07^b^	0.55 ± 0.71^ab^	0.18 ± 0.10	0.25 ± 0.06	0.25 ± 0.06
0.01	1.02 ± 0.64^y^	0.97 ± 0.80^y^	1.08 ± 0.65^y^	0.07 ± 0.07^β^	0.04 ± 0.03^β^	0.09 ± 0.03^β^	0.74 ± 0.51^a^	0.46 ± 0.42^b^	0.51 ± 0.24^ab^	0.23 ± 0.06	0.17 ± 0.02	0.27 ± 0.02
2	1.38 ± 0.20^y^	0.93 ± 0.45^y^	1.53 ± 0.77^y^	0.11 ± 0.05^αβ^	0.10 ± 0.07^αβ^	0.12 ± 0.08^αβ^	0.78 ± 0.67^a^	0.32 ± 0.23^b^	0.72 ± 0.19^ab^	0.36 ± 0.10	0.29 ± 0.13	0.29 ± 0.11
4	2.07 ± 0.07^x^	2.11 ± 0.33^x^	2.29 ± 0.53^x^	0.15 ± 0.1^α^	0.21 ± 0.03^α^	0.15 ± 0.03^α^	1.18 ± 0.04^a^	0.60 ± 0.34^b^	0.26 ± 0.08^ab^	0.17 ± 0.03	0.29 ± 0.02	0.31 ± 0.14

*Note*: ^a,b^Different superscripts within the same column indicate statistically significant differences among lactic acid values (*p* < .05). ^α,β^Different superscripts within the same column indicate statistically significant differences among malic acid values (*p* < .05). ^x‐y^Different superscripts within the same row indicate statistically significant differences among acetic acid values (*p* < .05).

Abbreviation: n.d., not detected.

The levels of organic acids in FFB may vary based on the fermenter culture. In the study by Bergqvist et al. (2005), two different LABs and their combinations resulted in significantly different concentrations of lactic acid. In the present study, tartaric acid was detected at low levels in the samples, and there was no significant ascorbic acid production (data not shown). Previously Seo et al. (2019) emphasized that the used strain of *L. brevis* had the ability to produce acetic acid, lactic acid, and succinic acid in MRS broth, whereas Zhang and Vadlani (2015) detected the production of lactic acid, acetic acid, and ethanol by *L. brevis*. However, since the amount of organic acid produced may vary based on the medium as well as the culture strain used, the results cannot be compared exclusively. Kun et al. (2008) also underlined the importance of medium composition, such as nitrogen and mineral contents, which impact the amount of lactic and acetic acids. On the other hand, the malic acid content increased significantly with the concentration of P4H2 (*p* < .05).

**FIGURE 3:**
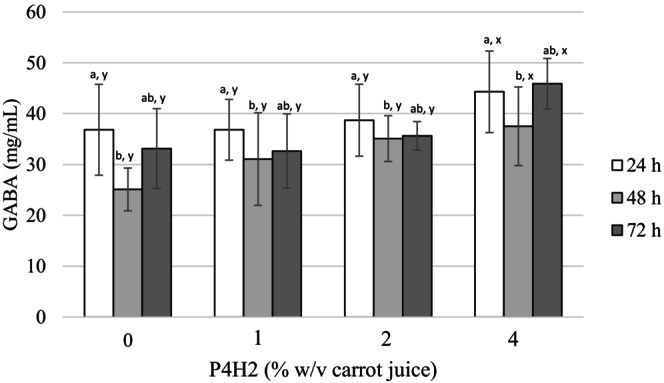
Gamma‐aminobutyric acid (GABA) concentration of FFBs throughout fermentation. ^a,b^Different superscripts within the different incubation times of same P4H2 concentration indicate statistically significant differences (*p* < .05). ^x,y^Different superscripts within the same incubation time of different P4H2 concentrations indicate statistically significant differences (*p* < .05).

Fermented functional beverages’ (FFBs') GABA concentration increased in correlation with pectin hydrolysate concentration, as seen in Figure 3. In particular, the amount of GABA in FFBs supplemented with P4H2 at 4% w/v carrot juice was found to be significantly higher than those in the other samples produced; additionally, it was higher than those in previous reports as well. The quantity of GABA in FFBs without pectin hydrolysate (fermented 24, 48 and 72 h) ranged between 25.09 and 36.83 mg/mL, whereas GABA was detected in the range of 37.51–45.89 mg/mL in FFBs including 4% P4H2. The highest amount of GABA (27.6 mg/mL) in black raspberry juice fermented with *L. brevis* GABA100 was observed on the 12th day at 30°C (Kim et al., 2009), which is lower than that given in the current study. While the produced strawberry juice with yeast extract included 262 mM GABA after fermentation with *L. brevis* CRL 2013 for 168 h (Cataldo, Villena, et al., 2020), tomato juice fermented with *Lactiplantibacillus plantarum* KB1253 resulted in 41 mM GABA. In another study, GABA synthesized by *Lactobacillus plantarum* DSM19463 in grape was around 4.83 mM after 72 h of fermentation (Di Cagno et al., 2010). Fermented mulberry juice with a co‐culture (*S. cerevisiae* SC125 and *L. plantarum* BC114) resulted in 2.42 mg/mL GABA (Zhang et al., 2020). Consequently, the GABA content achieved under the conditions of the current study was found to be significantly higher than those mentioned in other studies and the fermentation was completed at a shorter time interval as well. The *L. brevis* isolate used is not a universal culture and different isolation sources used in previous studies may cause differences in results as well. In another study, optimum conditions could not be achieved with *L. brevis* due to the fact that the fermentation environments were different (Falah et al., 2024).

Gamma‐aminobutyric acid (GABA), as a neurotransmitter, has important health effects on the nervous system (Diez‐Gutiérrez et al., 2020). Based on these results, it is predicted that the consumption of developed FFBs will have a significant health impact. Consumption of only 10 mg GABA daily by individuals with moderate hypertension for 12 weeks was found to be effective (Inoue et al., 2003). Moreover, Okada et al. (2000) mentioned that the intake of 26.4 mg GABA in rice germ daily had an impact on the treatment of neurological disorders. Besides, the Korean Ministry of Food and Drug Safety claims that consuming functional foods with a daily intake of 20 mg of GABA can effectively control blood pressure (Zhang et al., 2020). The fact that the amount of GABA contained in FFBs obtained by adding 2% MSG and pectin hydrolysate under 37°C, 72 h fermentation conditions was higher than these stated rates leads to the conclusion that it will have significant health effects.

### 3.4 Total phenolic content and antioxidant activity of FFBs

Fermentation may influence the antioxidant activity and total phenolic content of the products (Adebo & Gabriela Medina‐Meza, 2020). The changes in total phenolic content (TPC) and antioxidant activity in fermented carrot juice containing pectin hydrolysate depending on time and pectin hydrolysate content are shown in Table 6. The total phenolic contents of FFBs were detected in the range of 14.42–108.76 GE mg/g from the beginning of fermentation to the end of the 72‐h period. In particular, pectin concentration had a significant effect on the total phenolic component content (*p* < .05), and as its concentration increased, an increase in TPC was observed. Similarly, in the study of Valero‐Cases et al. (2023), high TPC was found when the concentration of inulin added as a prebiotic was the highest. According to several studies, pectin is a source of phenolic compounds, although it varies depending on the source from which it is obtained (Hosseini et al., 2020; Kumar et al., 2021).

**TABLE 6:** Total phenolic content and antioxidant activity of FFBs.

Analysis	P4H2 (%, w/v carrot juice)	Time (h)			
		0	24	48	72
Total phenolic content (GE mg/g)	0	16.36 ± 2.78^a,z^	14.42 ± 1.91^b,z^	15.21 ± 1.22^c,z^	16.84 ± 0.96^b,z^
	1	29.97 ± 1.28^a,y^	24.38 ± 2.59^b,y^	21.10 ± 3.27^c,y^	23.43 ± 3.58^b,y^
	2	58.45 ± 4.55^a,w^	51.24 ± 5.36^b,w^	46.98 ± 4.66^c,w^	48.31 ± 3.06^b,w^
	4	108.76 ± 10.83^a,x^	99.18 ± 6.19^b,x^	86.72 ± 15.57^c,x^	94.35 ± 6.87^b,x^
DPPH (TE mg/g)	0	5.28 ± 3.87^a,z^	6.21 ± 2.49^ab,z^	3.26 ± 4.09^b,z^	4.43 ± 3.77^b,z^
	1	27.68 ± 3.01^a,y^	23.75 ± 3.47^ab,y^	14.18 ± 7.73^b,y^	16.41 ± 5.6^b,y^
	2	41.65 ± 11.17^a,w^	40.94 ± 6.07^ab,w^	38.19 ± 5.84^b,w^	36.97 ± 6.43^b,w^
	4	120.53 ± 10.91^a,x^	110.86 ± 15.36^ab,x^	110.00 ± 17.03^b,x^	121.10 ± 21.69^b,x^
CUPRAC (TE mg/g)	0	57.03 ± 2.41^a,z^	44.02 ± 4.81^b,z^	40.7 ± 2.44^c,z^	36.45 ± 5.8^d,z^
	1	108.80 ± 8.54^a,y^	90.96 ± 7.91^b,y^	71.50 ± 20.62^c,y^	66.98 ± 16.48^d,y^
	2	217.77 ± 22.86^a,w^	180.00 ± 6.48^b,w^	162.49 ± 5.94^c,w^	156.8 ± 10.15^d,w^
	4	362.98 ± 14.3^a,x^	349.94 ± 10.4^b,x^	330.06 ± 9.82^c,x^	303.43 ± 13.05^d,x^

*Note*: ^a–d^Different superscripts within the same row indicate statistically significant differences (*p* < .05). ^v‐z^Different superscripts within the same column of analysis indicate statistically significant differences (*p* < .05).

While there is a decrease in TPC at the beginning of fermentation, there is no continuous decreasing trend depending on the fermentation time. An increase in TPC can be observed during fermentation, with the breakdown of various complex molecules into free or simpler compounds (Isas et al., 2020; Valero‐Cases et al., 2023). However, it is also observed that fermentation may cause a decline in TPC. Accordingly, Oh et al. (2017) reported a reduction in TPC of fermented blueberry with *Bacillus amyloliquefaciens*, *L. brevis*, and *Starmerella bombicola*, and the reason for this decline might be explained by the activity of microorganisms and produced enzymes that may convert phenolic substances into other bioactive molecules. Furthermore, variations in the fermenting culture's activity might be the primary cause of the discrepancies observed among studies (Ryu et al., 2019).

The antioxidant activity of FFBs was also evaluated by DPPH and CUPRAC methods (Table 6), and a trend similar to that seen in TPC was determined throughout fermentation. Although the effect of fermentation on TPC and antioxidant activity is similar, there was no correlation found between TPC and antioxidant activity (Isas et al., 2020). Similar to TPC, antioxidant activity showed statistical differences as pectin concentration increased (*p* < .05). Moreover, P4H2 added at 4% increased the values from 5.28 to 120.53 TE mg/g for DPPH, and from 57.03 to 362.98 TE mg/g for CUPRAC at the starting point of fermentation. Fermentation of apple juice increased TPC and antioxidant activity until a certain point of fermentation, after which there were slight declines (Li et al., 2018). Similarly, Kaprasob et al. (2017) observed a decrease in antioxidant activity with fermentation. However, the DPPH radical scavenging activity of fermented cashew apple juice increased up to the 12th hour of fermentation and then declined after the 48th hour. Throughout the fermentation in the current study, the antioxidant activities in 24, 48, and 72 h were investigated, and it might have been possible to obtain similar results with a fermented sample taken 24 h before. The decrease in the antioxidant activity was linked with the oxidation or degradation of related compounds (Kaprasob et al., 2017).

## 4 CONCLUSION

In the current study, it was aimed to produce a functional fermented beverage with the addition of pectin hydrolysate obtained from industrial pomegranate waste, where GABA production with *L. brevis* was aimed in particular. With 4% pectin hydrolysate and 72 h of fermentation at 37°C, 45.89 mg/mL of GABA was synthesized, the level of which was significantly higher than that reported previously. Based on these results, it is anticipated that the consumption of developed FFBs would have a noteworthy effect on consumers due to its high GABA content. Additionally, with the addition of pectin hydrolysate, an increase in both total phenolic content and antioxidant activity was observed, and a certain amount of lactic acid and malic acid production was obtained with fermentation. This will contribute to the production of a functional beverage with improvements in GABA content as well as other bioactive properties with fermentation, and an alternative product will be offered to consumers. With all these, it will be possible to evaluate the wastes generated at high volumes that can be a source of pectin and to support the zero waste policy. It is envisaged that this study can be improved in the future by monitoring fermentation with culture combinations or at intermediate periods. In addition, by expanding the GABA‐enriched functional food product category, it will be possible to utilize wastes rich in different prebiotics.

## AUTHOR CONTRIBUTIONS

**Dilara Devecioglu:** Data curation (equal); formal analysis (lead); validation (lead); visualization (lead); writing – original draft (equal). **Didem Kara:** Data curation (equal); investigation (equal). **Rabia Tapan:** Data curation (supporting); investigation (supporting). **Funda Karbancioglu‐Guler:** Conceptualization (equal); methodology (equal); project administration (equal); resources (equal); supervision (equal); writing – review and editing (equal). **Derya Kahveci:** Conceptualization (lead); funding acquisition (lead); methodology (equal); project administration (equal); resources (lead); supervision (equal); writing – original draft (equal); writing – review and editing (lead).

## FUNDING INFORMATION

This study was financially supported by the Scientific Research Council of Istanbul Technical University.

## CONFLICT OF INTEREST STATEMENT

The authors confirm that they have no conflicts of interest with respect to the work described in this manuscript.

## Data Availability

The authors confirm that the data supporting the findings of this study are available within the article. Raw data that support the findings of this study are available from corresponding author, upon reasonable request.
